# An Unusual Presentation of Acquired Perforating Dermatosis Confined to the Scalp

**DOI:** 10.7759/cureus.99636

**Published:** 2025-12-19

**Authors:** Agustina S Lambertini, Gabriela Montecchiesi, Valeria A Taboada, Máximo Lambertini, Andrea V Giuliani

**Affiliations:** 1 Department of Dermatology, Hospital Central de San Isidro, San Isidro, ARG; 2 Department of Medicine, Universidad Austral, Pilar, ARG

**Keywords:** acquired perforating dermatosis, allopurinol, chronic kidney disease, hemodialysis, pruritus, scalp, transepidermal elimination, type 1 diabetes mellitus

## Abstract

Acquired perforating dermatosis (APD) is a rare skin disorder characterized by transepidermal elimination of dermal connective tissue components. We report the case of a 31-year-old man with end-stage renal disease on hemodialysis and type 1 diabetes mellitus who presented with APD lesions confined to the scalp, an uncommon site for this condition. Histopathological examination confirmed the diagnosis, revealing transepidermal elimination of degenerated collagen bundles. Sequential treatment with oral clindamycin followed by allopurinol resulted in marked clinical improvement and complete resolution within one month of therapy.

## Introduction

Acquired perforating dermatosis (APD) is an uncommon skin disorder characterized by the transepidermal elimination of altered dermal connective tissue, including collagen and elastic fibers [1]. It represents part of a broader group of perforating disorders, which also encompasses Kyrle disease, perforating folliculitis, and reactive perforating collagenosis [2]. While these entities share the common mechanism of transepidermal extrusion, APD specifically refers to acquired forms associated with systemic diseases such as diabetes mellitus and chronic kidney disease (CKD) [3].

The exact pathogenesis remains unclear. Chronic microtrauma, oxidative stress, and impaired wound repair are thought to contribute to dermal necrosis and extrusion of connective tissue through the epidermis [4]. In diabetic and dialysis patients, microangiopathy, advanced glycation end-products, and uremic toxins further promote dermal degeneration and pruritus [5].

Clinically, APD manifests as intensely pruritic, umbilicated papules or nodules with a central keratotic plug, typically located on the extensor surfaces of the limbs and trunk [6]. Involvement of the scalp is exceptionally rare, with only isolated cases documented in the literature [6]. Beyond its clinical rarity, APD can significantly impair quality of life due to persistent itching and cosmetic discomfort. Reporting unusual presentations contributes to expanding the clinical spectrum of APD and may help refine therapeutic strategies, including emerging treatments such as apremilast and biologic agents [7].

## Case presentation

A 31-year-old man from Villa Adelina, Buenos Aires, with a history of hypertension and type 1 diabetes mellitus diagnosed at age five, presented with poor treatment adherence and progressive systemic deterioration. His comorbidities included bilateral proliferative retinopathy treated with panretinal photocoagulation, moderate axonotmesis affecting all four limbs, and end-stage CKD requiring thrice-weekly hemodialysis for the past five years.

**Figure 1:**
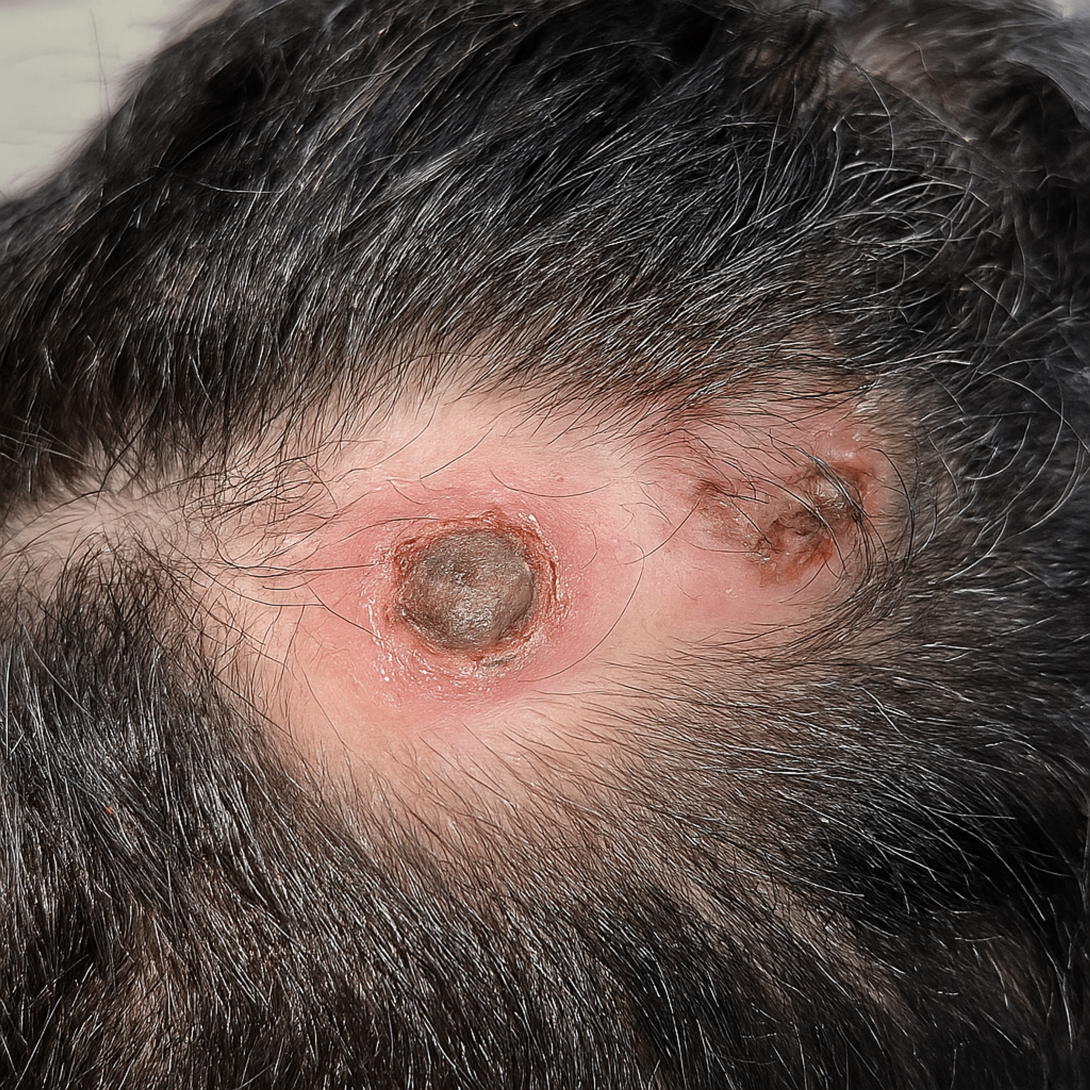
Crateriform nodule with a central keratotic plug on the scalp.

**Figure 2:**
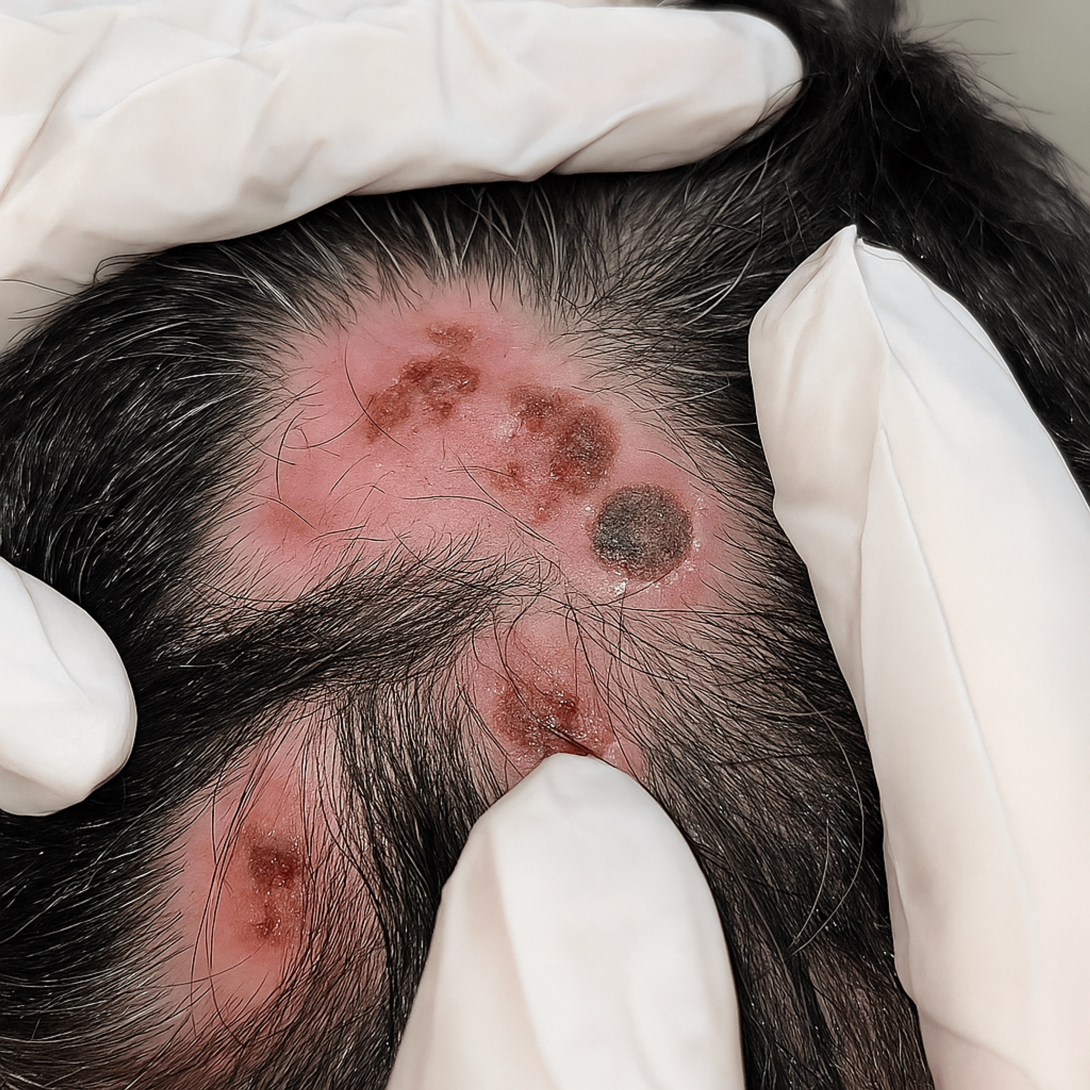
Multiple ulcerated and crusted nodules with central keratotic plugs over an alopecic plaque on the scalp.

He reported a two-year history of intensely pruritic dermatosis confined to the scalp. Physical examination revealed multiple umbilicated nodules averaging 1.5 cm in diameter, some ulcerated and others with central keratotic plugs. Lesions were tender to palpation and strictly limited to the scalp, with no involvement of other body sites (Figure 1, Figure 2). Based on the characteristic crateriform morphology with prominent central keratotic plugs, the main clinical differential diagnoses included prurigo nodularis and APD, particularly Kyrle disease and reactive perforating collagenosis. Although crateriform tumors such as keratoacanthoma may be considered in isolated lesions, their multiplicity, chronic course, and intense pruritus made a neoplastic process unlikely.

**Figure 3:**
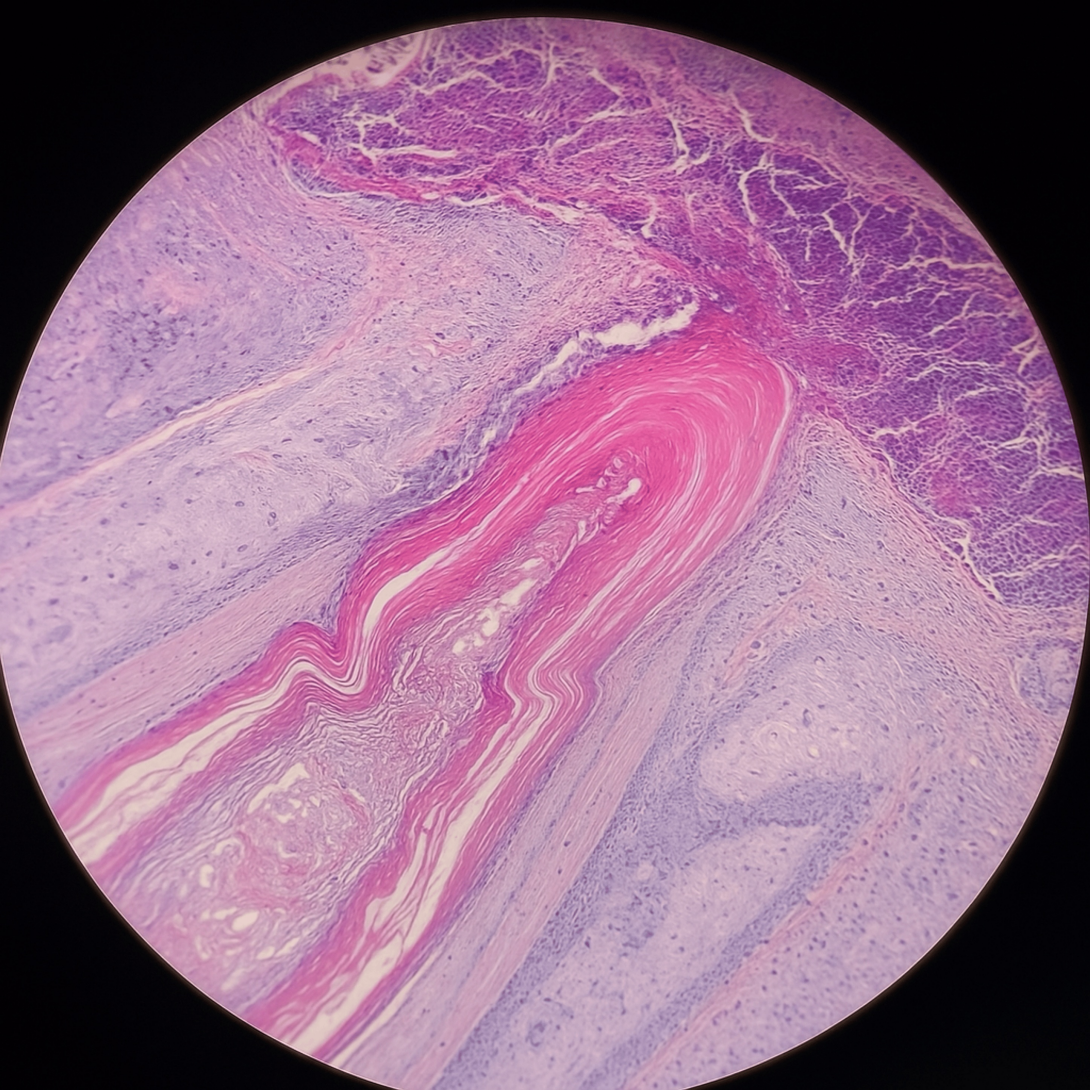
Dilated follicular infundibulum filled with abundant keratinous material.

Skin biopsies were obtained for histopathological examination and microbiological culture. The culture grew methicillin-sensitive Staphylococcus aureus. Histopathology demonstrated squamous epithelium with acanthosis and hyperkeratosis covered by a thick fibrin-leukocyte crust, a follicular infundibulum containing a central keratotic plug, and a perifollicular lymphoid infiltrate rich in neutrophils (Figure 3).

**Figure 4:**
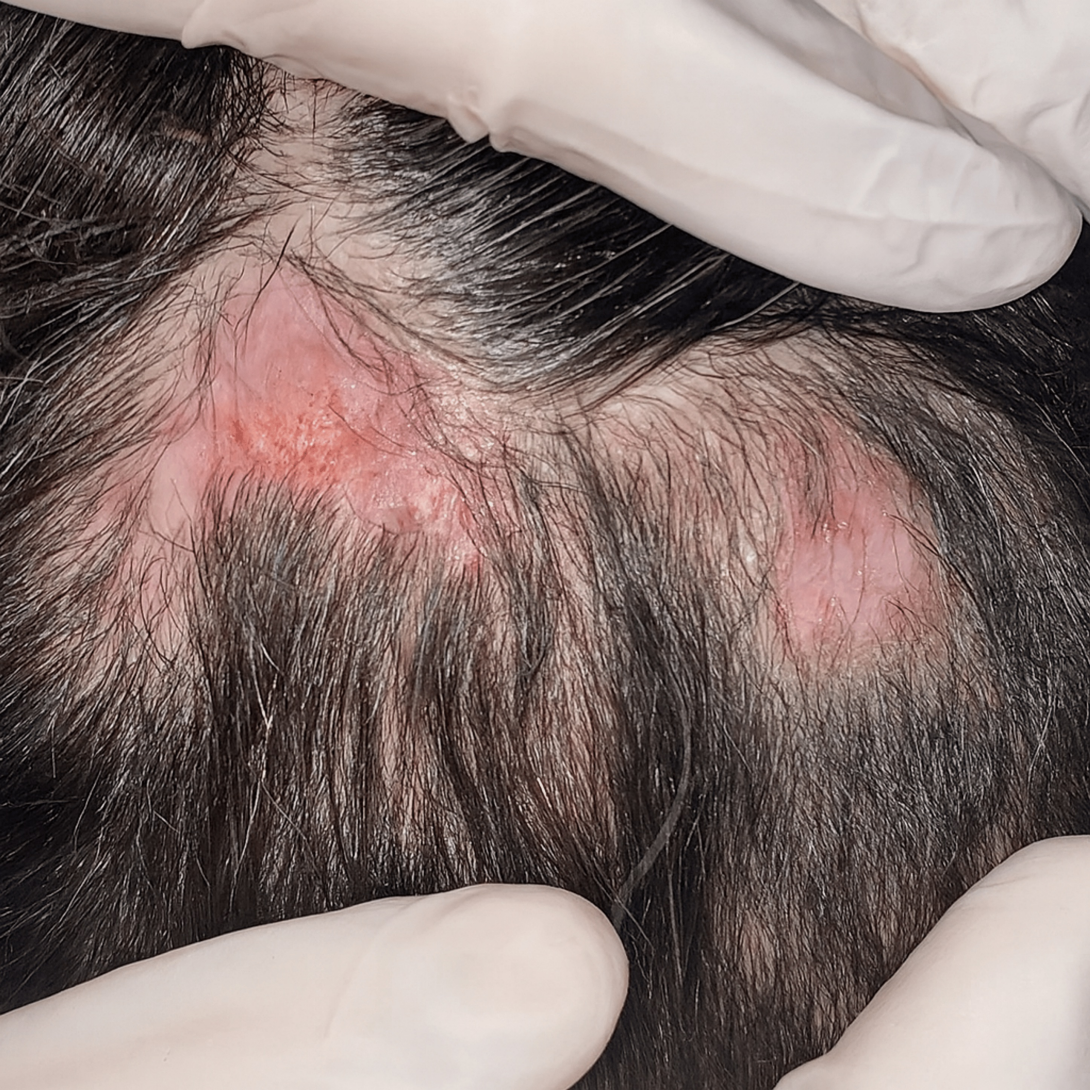
Re-epithelialization of the lesions.

Considering the clinical setting and histopathological findings, a diagnosis of APD was established. Initial management with topical fusidic acid and clobetasol resulted in partial improvement. Following nephrology consultation, oral clindamycin (300 mg every eight hours for three weeks) was initiated, leading to further resolution. Subsequently, oral allopurinol (100 mg/day) was added, achieving near-complete clearance of lesions after one month of therapy (Figure 4).

## Discussion

APD usually presents as umbilicated papules or nodules with a central keratotic plug and marked pruritus, often associated with the Koebner phenomenon [1]. Lesions most frequently affect the extensor surfaces of the limbs and trunk; exclusive scalp involvement, as observed in our patient, is exceptionally uncommon, with only a few cases reported [6].

The pathogenesis of APD is multifactorial. Chronic hyperglycemia in diabetic patients may lead to microangiopathy and collagen degeneration, while in CKD, the dermal deposition of calcium and glycation byproducts further contributes to tissue injury [3,5]. Repetitive scratching perpetuates trauma and transepidermal elimination [4]. Additionally, cytokine-mediated remodeling of the extracellular matrix has been proposed, involving Th2 cytokines such as IL-4, IL-13, and IL-31, and transforming growth factor β3 (TGF-β3), which promote epidermal proliferation and connective tissue extrusion [5].

Histopathological examination remains essential for diagnosis, showing a cup-shaped epidermal invagination filled with keratin, degenerated collagen, and inflammatory debris crossing the epidermis from the superficial dermis [8]. These findings distinguish APD from other follicular and perforating disorders, including Kyrle disease and perforating folliculitis [2,4].

Recent clinicopathologic studies have emphasized the diagnostic contribution of noninvasive techniques such as dermoscopy and reflective confocal microscopy, which reveal characteristic findings including central ulceration and peripheral loop-like vessels corresponding to transepidermal elimination zones [1]. These imaging modalities could facilitate earlier recognition of atypical APD presentations, such as scalp involvement.

Treatment focuses on controlling underlying systemic conditions, alleviating pruritus, and preventing infection. Traditional therapies, including topical corticosteroids, retinoids, keratolytics, and narrowband ultraviolet B phototherapy, have shown variable efficacy, often requiring long-term combination approaches and optimization of metabolic comorbidities [2]. In selected cases, localized anti-inflammatory treatment may be effective; Alshahrani et al. reported marked clinical improvement after a single session of intralesional triamcinolone in scalp-limited APD [6]. Our patient achieved near-complete resolution with oral clindamycin followed by allopurinol, consistent with previous reports demonstrating anti-inflammatory and antioxidative effects of these agents. Beyond pharmacologic therapy, Mima et al. described a favorable clinical response of acquired reactive perforating dermatosis following optimization of diabetes control and correction of peripheral vascular impairment [5].

Novel therapeutic approaches have emerged for refractory cases. Zaidi et al. described successful management of treatment-resistant APD with apremilast, a phosphodiesterase-4 inhibitor that downregulates inflammatory cytokines and improves pruritus [7]. Conversely, Alhadlg et al. reported APD induction in a patient receiving risankizumab, suggesting that biologic therapy may paradoxically alter cytokine signaling and trigger transepidermal elimination [8].

This case reinforces the importance of individualized management in APD and demonstrates how tailored therapy addressing both systemic and cutaneous components can lead to complete resolution, even in rare scalp-limited presentations.

## Conclusions

APD is a rare skin disorder that should be considered in patients with diabetes or CKD presenting with pruritic, keratotic papules or nodules. Although scalp involvement is extremely uncommon, its recognition is essential to avoid misdiagnosis and unnecessary treatments. Histopathology remains the cornerstone for confirmation, and management should focus on controlling systemic diseases, relieving pruritus, and preventing recurrent trauma. Our case highlights that oral clindamycin followed by allopurinol can be an effective therapeutic sequence, supporting the anti-inflammatory and antioxidative role of allopurinol in APD. Awareness of atypical presentations, such as scalp-confined lesions, broadens the clinical understanding of this entity and contributes to improved diagnostic and therapeutic outcomes.
